# Evidence of scavenging behaviour in crested porcupine

**DOI:** 10.1038/s41598-020-69252-z

**Published:** 2020-07-23

**Authors:** Francesca Coppola, Denise Guerrieri, Andrea Simoncini, Paolo Varuzza, Giuseppe Vecchio, Antonio Felicioli

**Affiliations:** 1 Department of Veterinary Sciences, University of Pisa, Pisa, Italy; 2 Wildlife Hunting Reserve “Camugliano”, Ponsacco, Italy; 3 Geographica Srl, Salerno, Italy; 4 Studio Agrofauna, Livorno, Italy

**Keywords:** Ecology, Zoology

## Abstract

The vegetarian diet of many herbivorous mammals is supplemented with proteins of animal origin, especially in young individuals and in breeding females, to provide key proteins necessary for both growth and breeding. Among porcupine species, only the Cape porcupine (*Hystrix africaeaustralis*) has been observed to consume carrion flesh. From June to August 2019, a pigeon carcass was placed together with corn in 7 study settlements and near 2 monitored capture-traps, in order to assess the carrion flesh feeding habits of the crested porcupine (*Hystrix cristata*)*.* Scavenging behaviour was recorded on four occasions. All the recorded individuals were adults and at least one was female. This demonstrates that the crested porcupine occasionally does eat flesh. Such evidence raises important questions concerning the relationship between feeding habits and the physiological needs of this herbivorous rodent.

## Introduction

Within Old world porcupines of the sub-genus *Hystrix* (*H. indica, H. africaeaustralis* and *H. cristata*), only *H. africaeaustralis* (Cape porcupine) and *H. cristata* (crested porcupine) show a sympatric distribution overlap in southeast Africa^1^, while *H. indica* (Indian porcupine) is widely distributed in semi-arid areas of south-western Asia^2^. The three species have thrived amidst anthropic activities, such as agriculture and manmade riverbanks, which provide rodents with enough food resources and burrowing habitats, respectively^3^. These rodents are infamous as pests for destroying economically important crops and damaging riverbanks by burrowing^4–6^.

All these species are monogamous, burrowing, mainly nocturnal rodents^7–11^ and spend most of their night-time hours searching for food^12–14^. The porcupine diet is mainly based on bulbs, tubers (also truffles), and roots, dug up with strong legs, but also includes flowers, fruits, grass, leaves, berries, barks, twigs, corns, and grains and seeds of wild and cultivated plants^15–18^. Porcupines usually partially eat their food, chewing it loudly and discarding the rest of the food after just a few mouthfuls^19^. Season, habitat richness and ecology strongly influence the porcupine diet^16,20,21^. Feeding habits change according to food availability and show wide ecological tolerance and fitness maximization^3^. All porcupines of the sub-genus *Hystrix* show osteophagia (i.e. gnaw the bones)^22,23^ and have long been known to collect bones inside and outside their burrows^24^. The reason for osteophagia in porcupines is still not clear. Honing of open-rooted incisors and/or calcium and phosphorous assumption are the two main hypothesis^20,22^. Porcupines have a simple stomach, a voluminous caecum^25^ and a peculiar furrow in the large intestine^26^. These anatomic features are compatible with caecotrophy practise although it has never been observed in porcupines^27^.

Of the three *Hystrix* species only the cape porcupine has been observed to eat carrion flesh^1,28^. Futhermore, Roth^28^ reported an 8–10 week old cape porcupine reared in captivity, that was fed on boiled ham and fried meat. Animal-based feeding behaviour has been reported also in *Hystrix indica*^29^, in *Atherurus africanus (*African brush-tailed porcupine, genus *Atherurus), Atherurus macrourus* (Asiatic brush-tailed porcupine, genus *Atherurus*)^29,30^ and in *Hystrix pumila* (Palawan porcupine, genus *Hystrix*, sub-genus *Thecurus)*^31^, although there is no direct observation. No data are available concerning this feeding habit neither in *H. cristata* nor in any of the new world porcupines.

Animal proteins in a vegetarian diet are a feature of frugivorous and granivorous rodents^32^. Animal proteins supplement the vegetarian diet of many herbivorous mammals, especially in growing youngsters and breeding females^33^. Therefore according to White^33^, otherwise vegetarian mammals actively looking for meat may be a far more common breeding strategy than has previously been presumed. The aim of this investigation was to assess whether animal proteins supplement the vegetarian diet of the crested porcupine as previously observed in the cape porcupine.

## Results

**Figure 1:**
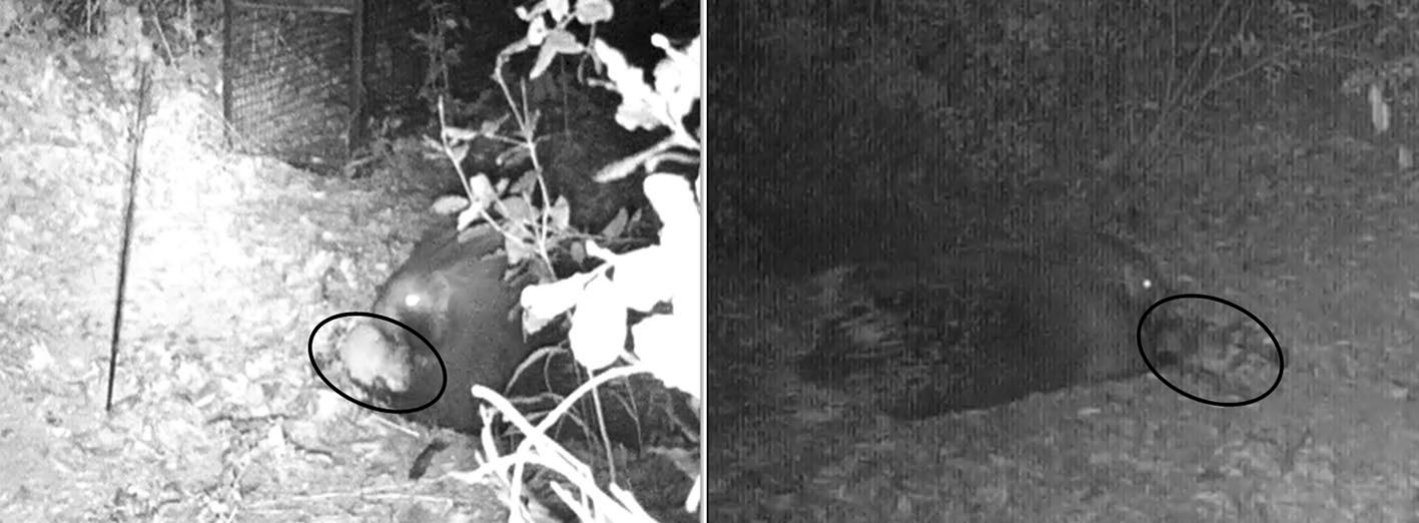
An unidentified adult porcupine of family 4 in settlement 7 (left) and the identified adult female porcupine of family 1 in settlement 1 (right) scavenging on a pigeon carcass (in black circle). Both the images are screenshots of videos recorded by camera-traps.

The average size of the family clans inhabiting the study settlements was 4.1 (SD = 1.6) individuals. All the study settlements were located in the woody ecotone strip within a maximum distance of 86.5 (M = 37.3, SD 22.7) meters from food-patch areas (i.e. cultivated or uncultivated zones), in sandy soil with a slope range from 5 to 10%. The average number of ground holes *per* settlement was 5.7 (SD = 4.5) holes. Five of the 7 monitored settlements were South-east/South-west oriented, while the other 2 were North-east/North-west oriented. Overall a total of four distinct scavenging events of a pigeon carcass by crested porcupines were observed (Fig. 1). The scavenging events were observed on one occasion in each of two settlements (S1 and S7, respectively) and on two occasions near 1 of 2 monitored capture-traps (T2). All the individuals observed to perform scavenging were adults and only one was certainly the adult female of family 1 (Table 1). In the events observed near the capture-traps the marking of the individuals could not be assessed. However, in one case it is plausible that it was the adult female of family 1. In one of the two events recorded near T2, a youngster was present with the adult porcupine without displaying an interest in the carcass. The same lack of interest was observed in the adult male present with the adult female of family 1. On four occasions two identified sub-adults, a male and a female of family 3, sniffed the pigeon but did not proceed to scavenge.

**Table 1:** Scavenging events of a pigeon carcass by crested porcupines recorded during the monitoring period. For each event the date, time, and site of observation and the age class, sex, and family of the focal porcupine are reported.

Events	Data	Time	Site	Age class	Sex	Family
1	13/06/2019	20:53	S1	Adult	Female	Family 1
2	05/06/2019	22:04	S7	Adult	Unidentified	Family 4
3	24/06/2019	01:45	T2	Adult	Unidentified	Unidentified
4	14/07/2019	23:20	T2	Adult	Unidentified	Unidentified

## Methods

### Study area and design

The study area of 514 ha was located in the Camugliano wildlife hunting reserve (10.64955 N–4359995 E) in Ponsacco-Capannoli, in the Province of Pisa (Tuscany, Central Italy). The area is characterized by low biodiversity and fragmentation, with a woody surface of about 100 ha evenly distributed. The woody cover is characterized by cops of *Quercus cerri, Q. pubescens* and *Q. ilex* while the undergrowth is mainly composed of *Ruscus aculeatus*. Wheat, sunflower, alfalfa, and vegetable crop areas, as well as vineyards and olive groves, surround the woody area. The cultivated areas are freely and easily accessible to wildlife through a network of pathways. The wildlife hunting reserve "Camugliano" supports pheasant breeding and hunting. Disposable mixed crops (chicory, forage cabbage, and sorghum) are annually sown to provide free food and shelter to pheasants. Corn is also provided daily throughout the year in artificial feeders located within the reserve. The mixed crops and corn are also a ready food source for crested porcupines throughout the year. In the study area 16 porcupine settlements (2.4 settlements/Km^2^) were known for a population density of 4 porcupines/Km^2^^34^.

**Figure 2:**
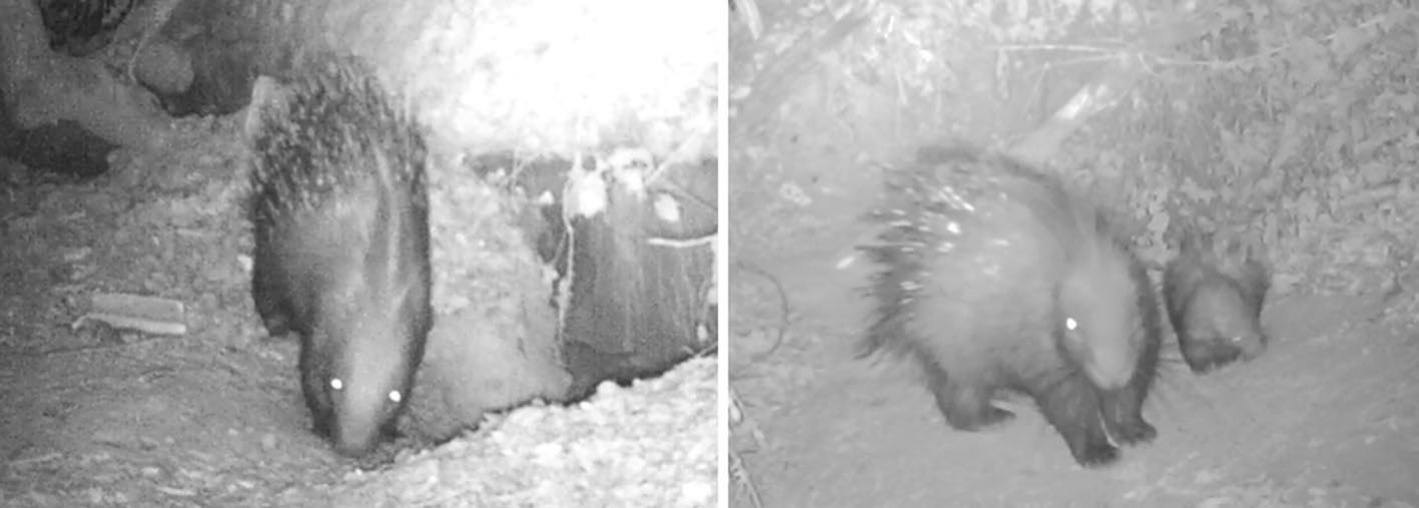
Porcupine with both eyes reflecting the light (left) and porcupine blind to the left eye (right) with a porcupette.

Between March and April 2019, a porcupine capture campaign in the vicinity of 7 study settlements, among the 16 known, was performed. Six capture-traps were placed in the porcupines’ most used pathways, being those pathways where signs of porcupine presence (quills and faeces) were detected on a weekly basis. All these pathways connected the inhabited settlements to the food patch-areas showing signs of new digs. All the study settlements and capture-traps were continuously monitored by camera-traps (Num’axes PIE1009) with passive infrared sensor (PIR) during the whole period of investigation. Each camera-trap was set to record 20 s of video each time it was triggered, without time-lapse and was active 24/7. The camera-traps’ video recordings were checked and filed on a weekly basis. Porcupines were trapped in wire mesh cages with a double entrance (110 × 42 × 42 cm), and baited with corn and potatoes. Porcupines’ capture and handling were carried out in accordance with the protocol approved by the Italian Institute for Environmental Protection and Research (ISPRA) and Tuscany Region. Captured porcupines were anesthetized, weighed, and sexed with age attributed according to weight^10^. Each captured porcupine was individually marked with coloured adhesive tapes applied to the quills, and/or by white or black paint sprayed on the crest and/or on the tail to support recognition in camera-trap videos. In addition, the individual recognition of some unmarked specimens was also possible due to the presence of phenotypic peculiarities such as blindness (Fig. 2), scars, and injuries. During the study, a total of eight porcupine individuals were identified, of which five were captured and marked, and three were identified according to recognizable phenotypic features. The individual recognition of these specimens allowed us to identify four different porcupine families inhabiting the monitored settlements (Table 2).

**Table 2:** Age class, sex, and recognisable features of the eight porcupines. For each recognisable porcupine the phenotypic peculiarity observed is reported.

Family	Age class	Sex	Recognisable features
Family 1	Adult	Female	Marked
	Adult	Male	Injury in the rump (left) Blindness of the right eye
Family 2	Adult	Female	Marked
Family 3	Sub-adult	Female	Marked
	Porcupette	Female	Marked
	Sub-adult	Male	Injuries in the rump (left)
Family 4	Adult	Male	Blindness of the right eye
	Sub-adult	Female	Marked

**Figure 3:**
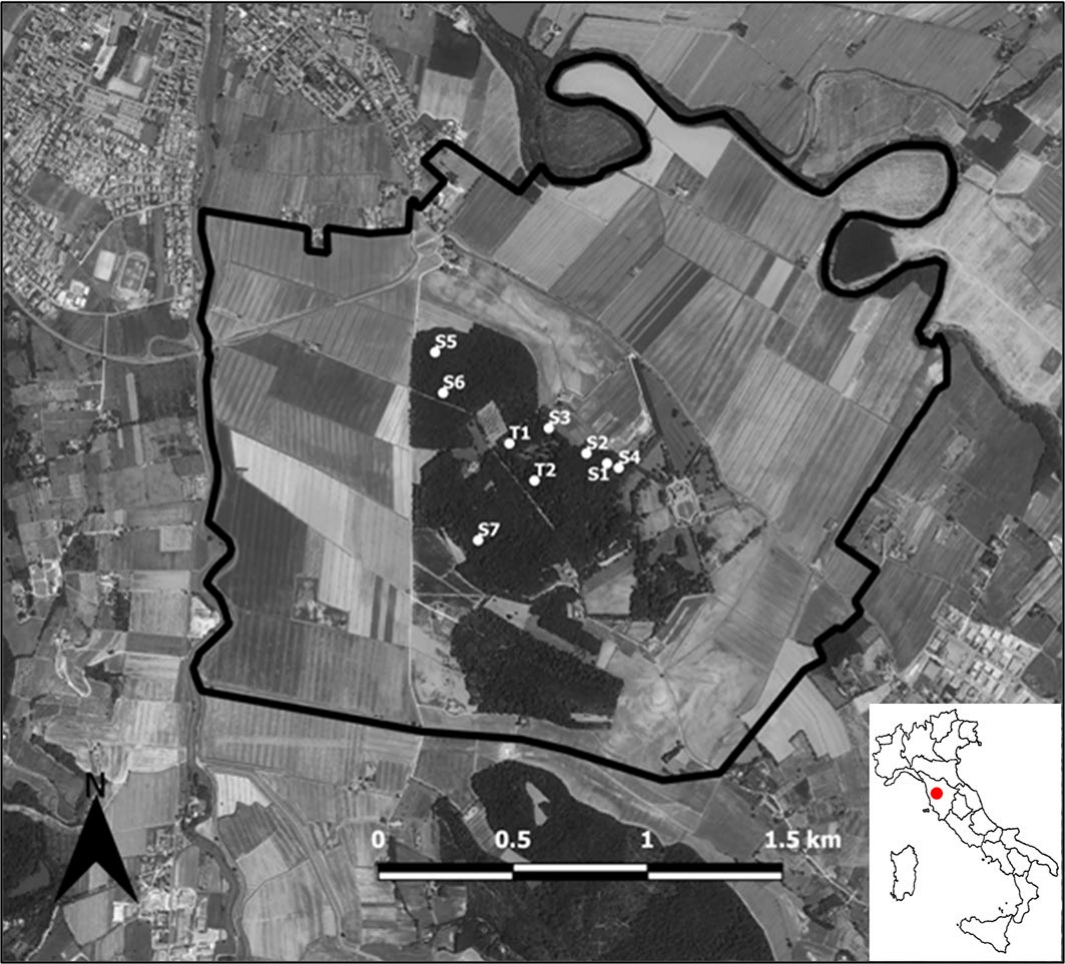
The Camugliano wildlife hunting reserve (border black line) where the investigation was performed. The white dots indicate the 7 study settlements (S1 to S7) and the 2 capture-traps (T1 and T2) where pigeon carcasses and corn were both provided. The location and country map are visible in the inset. The study area image was created using QGis 2.18 software (https://www.qgis.org/it/site/).

**Figure 4:**
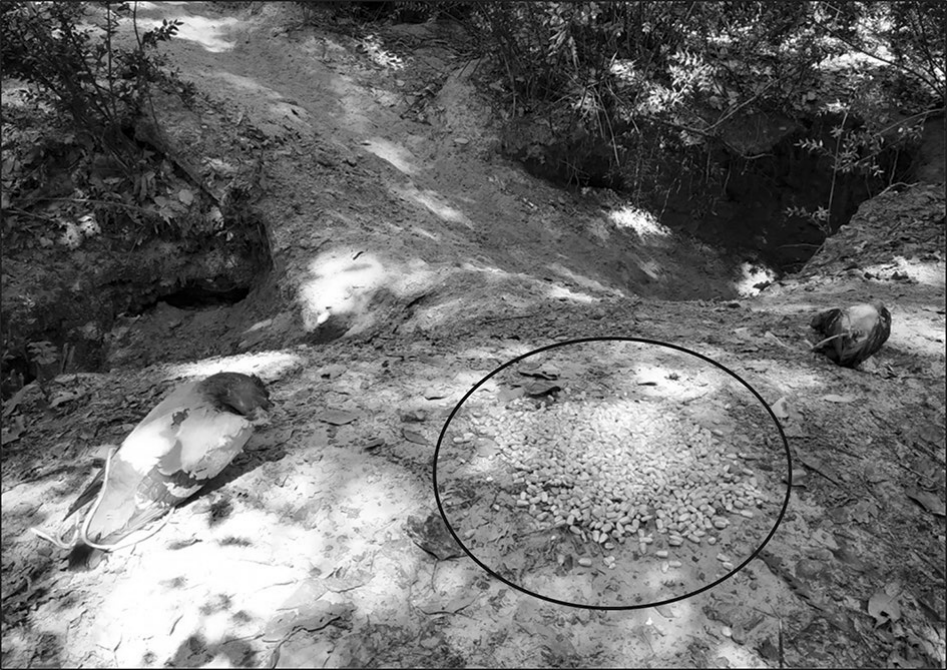
Setup of both pigeon carcasses and corn (in black circle) placed in settlement 7.

From June to August 2019 a pigeon carcass and 500 g of corn were placed in each of the 7 monitored settlements (S1–S7) and in the vicinity of two out of six monitored capture-traps (T1–T2) (Fig. 3 and Fig. 4). Pigeon carcasses were obtained from authorized programmed culling performed in the Camugliano wildlife hunting reserve for agricultural crops damage control.

Every 2 days, the pigeon carcasses were replaced and the camera traps’ videos were checked. Scavenging of pigeons by porcupines was assessed when tearing of meat from the carcass could be clearly observed. For each pigeon scavenging event, the date, time and, whenever possible, age class and sex of the individuals were recorded. Unmarked porcupettes and youngsters up to 5–6 months old were identified on the recorded camera-traps’ videos, by their smaller body sizes. Conversely, youngsters over 7 months old, thereby with a body size similar to adults, were identified only when both adults of the family were individually recognisable. Whenever porcupine age class identification was not possible, the animals were attributed as adults.

### Ethical approval

The capture-marking procedures performed during the study were approved by the Italian Institute for Environmental Protection and Research (ISPRA) [Protocol No.:150071; Dated: 16 March 2018] and by Tuscany Region [Decree n. 4842; Dated: 6 April 2018]. The procedure for pigeon containment was authorized by the Province of Pisa, according to art. 37, L.R.T. 3/94 [Intervention code 113; Dated: 4 February 2019].

## Discussion

This investigation confirms carrion scavenging behaviour in crested porcupines (*H. cristata*) for the first time. Among the old world porcupines, such scavenging behaviour has been previously observed and described only in Cape porcupines^1,28^. Feeding on carrions has also been reported, although not directly observed, in *H. indica, Atherurus macrourus*^29^, *Atherurus africanus*^30^ and in *Hystrix pumila*, the latest reported as the most common Palawan carnivore^31^ (Table 3). To the best of our knowledge the scavenging behaviour seems not to be reported in new world porcupines.

**Table 3:** Comparative table of the available scavenging behaviour data in old world porcupines. For each species the type of data and source are reported.

Presence of scavenging behaviour in old world porcupine				
	Type of data		Literature source	
**Genus** ***Hystrix***				
*H. africaeaustralis*	Observed		Roth^28^ Kingdon^1^	
*H. cristata*	Reported		Hutchins et al.^29^	
*H. indica*	Reported		Hutchins et al.^29^	
*H. pumila*	Reported		Marler et al.^31^	
**Genus** ***Atherurus***				
*A. africanus*	Reported		Jori et al.^30^	
*A. macrourus*	Reported		Kleiman et al.^29^	

The scavenging behaviour of crested porcupine reported here, was recorded in a habitat characterized by high food availability. Moreover, the presence of both pigeon and corn during all the scavenging events recorded, indicates a potential preference for flesh by porcupines, at least on these occasions. Therefore, feeding on carrion flesh by crested porcupine is not related to a lack of food and is more likely due to physiological needs. A primarily vegetarian diet supplemented with animal protein is common in many frugivorous and granivorous rodents, especially in growing young and breeding females^32,33^. Integration of animal protein into otherwise vegetarian diets seems to be the result of behavioural and physiological adaptations to increase reproductive success^33^. In this investigation, feeding on carrion flesh was clearly observed only in adult specimens, at least one of which was certainly a female. The observation of this feeding behaviour in a porcupine female suggests that eating animal protein could be a feeding strategy for the assumption of key proteins necessary for breeding as suggested by White^33^. Scavenging behaviour by porcupines could also be a route to infection of zoonotic diseases. The recent detection of *Giardia duodenalis*^35^, several *Leptospira* serogroups^36^ and the isolation of *Leptospira* serovar Pomona^37^ in crested porcupines supports this possibility. Further investigations are necessary how scavenging behaviour in crested porcupines relates to age, sex, and physiological state.

## Supplementary information

Supplementary information 1.

Supplementary information 2.

## Data Availability

All data are available on request to the corresponding author.

## References

[1] Kingdon J, Kingdon J (1974). Porcupines (*Hystrix*). East African Mammals.

[2] Arslan A (2008). On the indian crested porcupine, *Hystrix indica* (Kerr, 1972) in Turkey (Mammalia: Rodentia). Pak. J. Biol. Sci..

[3] Pillay KR, Wilson AL, Ramesh T, Downs CT (2015). Digestive parameters and energy assimilation of Cape porcupine on economically important crops. Afr. Zool..

[4] Alkon PU, Saltz D (1985). Potatoes and the nutritional ecology of crested porcupine in a desert biome. J. Appl. Ecol..

[5] Khan AA, Mian A, Hussain R (2014). Deterioration impact of Indian crested porcupine, *Hystrix indica*, on irrigated forest plantations in Punjab Pakistan. Pak. J. Zool..

[6] Laurenzi A, Bodino N, Mori E (2016). Much ado about nothing: assessing the impact of a problematic rodent on agriculture and native trees. Mammal Res..

[7] Felicioli A, Grazzini A, Santini L (1997). The mounting behaviour of a pair of crested porcupine *Hystrix cristata* L. Mammalia.

[8] Van Aarde RJ (1987). Reproduction in the Cape porcupine *Hystrix africaeaustralis:* an ecological perspective. S Afr. J. Sci..

[9] Sever Z, Mendelssohn H (1988). Copulation as a possible mechanism to maintain monogamy in porcupines *Hystrix indica*. Anim. Behav..

[10] Coppola F, Vecchio G, Felicioli A (2019). Diurnal motor activity and “sunbathing” behaviour in crested porcupine (*Hystrix cristata* L., 1758). Sci. Rep..

[11] Coppola, F., Dari, C., Vecchio, G., Scarselli, D. & Felicioli, A. Co-habitation of settlements between Crested Porcupines (*Hystrix cristata*), Red Foxes (*Vulpes vulpes*) and European Badgers (*Meles meles*). *Curr. Sc *(in press).

[12] Alkon PU, Saltz D (1988). Foraging time and the northern range limits of Indian crested porcupines (*Hystrix indica* Kerr). J. Biogeo..

[13] Santini, L. The habits and influence on the environment of the old world porcupine *Hystrix cristata* L. in the northernmost part of its range. In *Proceedings of the 9th Vertebrate Pest Conference* 34, 149–153 (1980).

[14] Sever Z, Mendelssohn H (1991). Spatial movement patterns of porcupines (*Hystrix indica*). Mammalia.

[15] Barthelmess EL (2006). *Hystrix africaeaustralis*. Mamm. Species.

[16] Bruno E, Riccardi C (1995). The diet of the Crested porcupine *Hystrix cristata* L., 1758 in a Mediterranean rural area. Z. Saugetierkd..

[17] Hafeez S, Khan GS, Ashfaq M, Khan ZH (2011). Food habits of the Indian crested porcupine (*Hystrix indica*) in Faisalabad Pakistan. Pak. J. Agric. Sci..

[18] Ori F, Trappe J, Leonardi M, Iotti M, Pacioni G (2018). Crested porcupine (*Hystrix cristata*): mycophagist spore dispersers of the ectomycorrhizal truffle Tuber aestivum. Mycorrhiza.

[19] Chevallier, N. & Ashton, B. A report on the porcupine quill trade in South Africa. Preprint at https://media.withtank.com/23fd88d381/porcupine_quill_trade (2006). Accessed 5 February 2020.

[20] Akram F, Ilyas O, Haleem A (2017). Food and feeding habits of Indian crested porcupine in Pench Tiger Reserve, Madhya Pradesh India. Amb. Sci..

[21] Oussou CTB, Mensah GA, Sinsin B (2006). Etude de l’écologie du porc-épic (*Hystrix cristata*) et de son régime alimentaire en milieu naturel. BRAB.

[22] Duthie AG, Skinner JD (1986). Osteophagia in the Cape porcupine *Hystrix africaeaustralis*. S. Afr. J. Zool..

[23] Kiibi JM (2009). Taphonomic aspects of African porcupines (*Hystrix cristata*) in the Kenyan Highlands. J. Taphon..

[24] O’Regan HJ, Kuman K, Clarke RJ (2011). The likely accumulators of bones: five Cape Porcupine Den assemblages and the role of porcupines in the post-member 6 infill at Sterkfontein South Africa. J. Taphon.

[25] Van Jaarsveld AS, Knight-Eloff AK (1984). Digestion in the porcupine *Hystrix africaeaustralis*. S. Afr. J. Zool..

[26] Gorgas M (1967). Comparative anatomical studies on the gastrointestinal tract of Sciuromorpha, Hystricomorpha and Caviomorpha (Rodentia). Z. wiss. Zool..

[27] Hagen KB, Hammer S, Frei S, Ortmann S, Glogowski R, Kreuzer M, Clauss M (2019). Digestive physiology, resting metabolism and methane production of captive Indian crested porcupine (*Hystrix indica*). J. Anim. Feed Sci..

[28] Roth HH, Mohr E, Rohrs M (1964). Note on the early growth development of *Hystrix africaeaustralis*. Zeitschrift fur saugetierkunde.

[29] Hutchins M, Kleiman DG, Geist V, McDade MC (2003). Grzimek’s Animal Life Encyclopedia.

[30] Jori F, Lopez-Bejar M, Houben P (1998). The biology and use of the African brush-tailed porcupine (*Atherurus africanus*, Gray, 1842) as a food animal A review. Biodivers. Conserv..

[31] Marler, P. N., Castro, L. S. G. & Hoevenaars, K. 2015. Mammalian Fauna of the proposed Cleopatra’s Needle Forest Reserve (CNFR): A Camera Trap Study of Palawan’s Mammals. In *Proceedings of the 2nd Palawan Research Symposium 2015,* 87–93 (2015).

[32] White TCR (2007). Mast seeding and mammal breeding: can a bonanza food supply be anticipated?. N.Z. J. Zool..

[33] White TCR (2011). The significance of unripe seeds and animal tissues in the protein nutrition of herbivores. Biol. Rev..

[34] Coppola, F. New knowledge tools for crested porcupine (*Hystrix cristata* L., 1758) management in the wild. First census model, new behavioural ecology aspects and preliminary investigation on health status. Ph.D. thesis (2020).

[35] Coppola F, Maestrini M, Berrilli F, Procesi IG, Felicioli A, Perrucci S (2020). First report of *Giardia duodenalis* infection in the crested porcupine (*Hystrix cristata* L., 1758). Int. J. Parasitol. Parasites Wildl..

[36] Coppola F, Cilia G, Bertelloni F, Casini L, D’Addio E, Fratini F, Cerri D, Felicioli A (2020). Crested Porcupine (*Hystrix cristata* L.): a new potential host for pathogenic leptospira among semi-fossorial mammals rested porcupin. Comp. Immunol. Microbiol. Infect. Dis..

[37] Cilia G, Bertelloni F, Coppola F, Turchi B, Biliotti C, Poli A, Parisi F, Felicioli A, Cerri D, Fratini F (2020). Isolation of *Leptospira* serovar Pomona from a crested porcupine (*Hystrix cristata*, L., 1758). Vet. Med. Sci..

